# 

**Published:** 1915-10-30

**Authors:** 


					THE RED CR0S5 AND ST. JOHN'S
ORGANISATION.
In our article on the above, on page 86 of last
week's issue, in the eleventh line of the second para-
graph in the second column, the figure 105 should be
1,050. The clause would then read " the 4,000 buildings
offered privately for auxiliary hospitals have been
inspected and reported on, of which 1,050 have been
formally accepted by the War Office."
110 THE HOSPITAL October 30, 1915.
PASSING EVENTS AND TOPICS.
Medical Students at
The games clubs in connection with Guy's Hospital
have resumed their activities, and we agree with the
Editor of the Gazette that there is every reason why men
who remain at the hospital should consider it their duty
to maintain physical fitness as well as to improve their
medical efficiency, since both are so necessary in the future
service of their country.
Game for the Wounded.
The appeal for game for the wounded soldiers in London
hospitals has met with a fairly generous response, and
during the last two months the game received has included
nearly 3,000 brace of grouse, partridges, and pheasants, and
many haunches of venison. We hope sportsmen and others
will continue to bear the London hospitals in mind and
make a practice of sending at least a good part of their
gifts to Mr. T. Comyn Piatt, 1900 Club, 3 Pickering Place,
London, S.W., through whom arrangements have been
made for distributing the game to the various establish-
ment t,.J
Surgery Hours and Dark Nights
It has been suggested to the Panel Committee of
London that in view of the earlier closing of chemists'
shops on account of further restrictions in the Lighting
Order, medical practitioners should be urged to adopt
earlier evening hours of consultation. Practitioners on the
panel, however, in the various1 areas have to be in attend-
ance at a time as far as possible convenient to the insured
persons in those areas. It is hardly probable that the Com-
mittee will desire to offer any general expression of opinion
which might appear to interfere with the judgment of
individual practitioners on this matter.
The New Insurance Drug Tariff.
There now appears to be little doubt that panel chemists
will continue service under the Insurance Act when the
proposed new tariff comes into force. This notwithstand-
ing the fact that they consider the rates allowed in respect
of establishment charges to be too low. On Thursday
last week, however, Mr. Charles Roberts, M.P., the
administrative head of the Insurance Act, promised a
deputation of panel chemists that if satisfactory evidence
were produced to show that the rate should be raised the
Commissioners would act on this evidence. It still re-
mains to be seen whether the medical profession will agree
to the adoption of a new tariff which would make the cost
of drugs the first charge on the Medical Benefit Fund.
Sanitary Inspectors in Conference.
Like all other public bodies which hold annual confer-
ences the Sanitary Inspectors' Association has been com-
pelled by the circumstances of the time to depart from
the plans originally made for this year's meeting. Owing
to the war last year's meeting was abandoned, and for
the fiame reason the conference this year had a dura-
tion of two days instead of the usual week, and was
unaccompanied by the usual visits and hospitalities; it
was held on October 29 and 30 at Carpenters' Hall,
London Wall, E.C., and the Presidential Address was
delivered by Sir James Cricliton-Browne. Two of the
subjects to be considered were " Sanitation and the Indi-
vidual" and " State Control of Sanitary Administration,"
both of which gave promise of useful and informative
discussions.
Farmers and the Red Cross.
In appealing to the farmers of East Sussex to subscribe
more liberally to the funds of the Red Cross, Mr. Her-
bert Brown gave to a meeting at Lewes a few particu-
lars of what farmers had done in other parts of the
country. At Leicester, for instance, forty-two fat beasts
were given, and altogether something like 600 to 700
head of cattle, which realised ?3,000. The British
farmers have already got four hospitals?two in Serbia,
one in Belgium, and the fourth in Egypt. In addition
they have provided fifty motor-ambulances and sent
?20,000 to the Dardanelles. In all about ?100,C03 has
been collected in six months.
Sir A. Wright's Newest Ideas.
At the Royal Society of Medicine's recent Exhibition
of Fracture Apparatus a paper was read by Sir Almroth
Wright, C.B., F.R.S., embodying his latest researches
on the irrigation of wounds by means of bandages used
a3 conductors of therapeutic solutions. These ideas, as
Sir A. Wright's always are, contain many very interest-
ing applications of the principles of osmosis and siphonage.
The apparatus is usually of very simple construction, and
the lecture is a model of terse and rational argument.
It will be of the greatest interest to see whether these
methods prove to be of the far-reaching use in practical
working that Ave must all hope they may be.
War Lectures
A Chadwick Lecture was delivered on October 29,
at the premises of the Royal Society of Medicine, 1 Wim-
pole Street, Cavendish Square, London, W., by Dr-
R. 0. Moon. The subject was " The Relation
of Typhus to (?) Plague, (b) Relapsing Fever,
(c) Enteric Fever," this being the second of the series on
Typhus in Serbia. Dr. Moon is physician to the Serbian
Isolation Hospital at Skoplje (Uskub), and therefore
speaks authoritatively, and with first-hand knowledge,
on the subject. He will deliver the third of the series
on November 3. Another lecture is announced for
November 10, at 8.15 p.m., at the Royal Institute of
British Architects, Conduit Street, W., the subject being
" Emergency Military Hospital Construction," and the
lecturer Mr. A. Saxon Snell, F.R.I.B.A.
An Appeal for a Battersea Dispensary.
A special appeal has been issued by the Mayor of
Battersea, the Vicar, and the leaders of the Nonconformist
and Roman Catholic communities, for support to the funds
of the Battersea Dispensary for the Prevention of Con-
sumption. The dispensary's ideal is a double one
?that there should not be an uncared-for case of tubercu-
losis in the borough, and that there shall be no house
left without medical supervision in which a case ha?
occurred. The statistics show that 2,644 patients have
been examined at the dispensary or at their own homes;
19,790 attendances have been made at the dispensary >
6,716 visits have been paid to the homes of the patients
by the doctor and the nurse attached to the dispensary, i?
addition to the visits made by voluntary workers; 244
patients too ill to attend the dispensary, most of whom
were referred to the dispensary by private doctors or
hospitals, have been treated in their own homes for vary-
ing periods by its staff, and . 455 patients have been sent
to hospitals for advanced cases, to sanatoria, or to insti-
tutions for operation or special treatment.
IUtis or Sebscbiption ; Twelve months, 6/6; Six months, 4/-; Three months, 2/-, post free to any address in the United Kingdom.
Abroad, Twelve months, 8/8; Six months, 5/-; Three months, 2/6, post free.?Address The Subscription Dept., "The Hospital,"
The Hospital Building, 28/29 Southampton Street, Strand, London, W.C.

				

